# Sugar feeding by *Aedes albopictus* in the Torres Strait, Australia

**DOI:** 10.1371/journal.pntd.0012856

**Published:** 2025-02-07

**Authors:** Boni F. Sebayang, Tanya L. Russell, Susannah Mosby, Richard Gela, Darcy L. Roeger, Bram van de Straat, Kyran M. Staunton, Thomas R. Burkot

**Affiliations:** 1 College of Public Health, Medical and Veterinary Science, Australian Institute of Tropical Health and Medicine, James Cook University, Smithfield, Queensland, Australia; 2 Community Services Department, Torres Strait Island Regional Council, Masig (Yorke) Island, Queensland, Australia; 3 Community Services Department, Torres Strait Island Regional Council, Hammond Island, Queensland, Australia; 4 Operational Science and Surveillance Group, Biosecurity Plant Division, Department of Agriculture, Fisheries and Forestry, Cairns International Airport, Cairns, Queensland, Australia; University of Queensland & CSIRO Biosecurity Flagship, AUSTRALIA

## Abstract

**Background:**

The establishment of *Aedes albopictus* in the Torres Strait Islands in 2005 is a threat to dengue control in the islands and on mainland Australia. Attractive targeted sugar baits (ATSBs) have been proposed as a control strategy for outdoor mosquitoes like *Ae. albopictus*. The sugar feeding behaviours of *Ae. albopictus* was studied to ascertain the potential of ATSBs to mitigate the risk of *Ae. albopictus* invading mainland Australia from the Torres Strait Islands.

**Methodology/Principal Findings:**

*Aedes albopictus* was sampled by sweep net collections in village and bushland habitats across two islands both in the mornings and afternoons. Samples were analysed to determine adult abundance as well as fructose prevalence and content by cold-anthrone test. Sampling stations were characterised by vegetation surveys and included the prevalence of flowers and fruit, and canopy cover. Among the 6,186 captured *Ae. albopictus*, the prevalence of fructose was 31.6% ± 1.4 in males and 30.5% ± 1.2 in females, with fructose averaging 62.9 µg (± 1.4) in fructose-positive *Ae. albopictus*.

**Conclusions:**

Mosquito sex and collection time were associated with the abundance of *Ae. albopictus* as well as fructose prevalence and content in *Ae. albopictus*. Male and female *Ae. albopictus* exhibited sugar abundance and prevalence comparable to studies where ATSBs were effective suggesting that ATSBs could potentially reduce *Ae. albopictus* populations in the Torres Strait Islands.

## Author summary

The establishment of *Ae. albopictus* in the Torres Strait Islands poses an ongoing challenge to dengue control in these islands. Additionally, these mosquitoes threaten to spread to mainland Australia. Baseline data on fructose prevalence in *Ae. albopictus* were thereby collected to ascertain the potential of attractive targeted sugar baits (ATSBs) to control *Ae. albopictus* populations and to mitigate both the risk of local dengue transmission and the spread of *Ae. albopictus* to mainland Australia. Using sweep net sampling, 6,186 *Ae. albopictus* mosquitoes were collected on the Hammond and Yorke Islands. Of these, 24–46% of males and 24–32% of females harboured detectable fructose, averaging 62.9 µg fructose per sugar positive mosquito. The abundance, fructose prevalence and fructose content of *Ae. albopictus* differed by mosquito sex and collection times. The fructose prevalence observed were comparable to those in vector populations that were significantly reduced by ATSBs elsewhere.

## Introduction

The Torres Strait Islands, between north Queensland, Australia, and Papua New Guinea, were historically inhabited by *Aedes aegypti*. *Aedes albopictus* was first detected in the Torres Strait in 2005 and has since displaced *Ae. aegypti* across the outer islands [1]. Microsatellite loci and mitochondrial COI sequences of *Ae*. *albopictus* suggest a likely introduction from Indonesia [2].

*Aedes albopictus* and *Ae*. *aegypti* are both vectors of dengue, Zika and chikungunya viruses [3,4]. *Aedes aegypti* is highly anthropophagic, endophagic and endophilic and oviposits in artificial containers [5]. *Aedes albopictus*, on the other hand, is predominantly exophagic, feeds on a wide range of host blood meal sources, is exophilic and oviposits in both natural and artificial containers [6]. Both species are highly invasive, with their migrations facilitated by ovipositing desiccation-resistant eggs in artificial containers that are transported on planes and ships [7]. Vector control is essential to curb transmission of the arboviruses spread by these vectors and to prevent its dispersal [1,8].

The establishment of *Ae*. *albopictus* across the Torres Strait Islands directly threatens dengue control on mainland Australia [9]. Firstly, by introducing a competent dengue vector to north Queensland where *Wolbachia-*infected *Ae*. *aegypti* had replaced the original dengue competent non-*Wolbachia* infected *Ae. aegypti* [10]. Further, cold-tolerant *Ae. albopictus* could expand the geographic range where dengue could be transmitted to include all major capitals cities which are also free from *Ae*. *aegypti* [9].

In the Torres Strait Islands, initial efforts to eliminate *Ae*. *albopictus* using adult and larval control failed [11]. Adult control was based on outdoor fogging which is expensive and requires multiple insecticide applications by a large workforce. Larval control is challenging to implement effectively due to the abundance of artificial and natural habitats, many of which are cryptic and thus difficult to find and treat. Elimination was not achieved and due to high implementation costs and workforce requirements the program was realigned [11]. In 2009, a cordon sanitaire was established on Horn and Thursday Islands (the major travel hubs in the Torres Strait) with the goal of preventing *Ae. albopictus* dispersing via these travel hubs to become established in Australia [11]. Intensive vector surveillance coupled with adult and larval control established an *Ae. albopictus* free zone in the cordon sanitaire.

In spite of intensive control within the cordon sanitaire, incursions of *Ae. albopictus* continue to be detected [12]. However, implementing control of *Ae. albopictus* in the outer islands might reduce the risk of ongoing incursions into the cordon sanitaire and correspondingly reduce the risk of its spread to mainland Australia. Effective control strategies for *Ae. albopictus* need to be based on the local *Ae*. *albopictus* biology, and to be simple and easy to deploy in the remote outer Torres Strait Islands where there is minimal infrastructure and capacity [13].

Recently, attractive targeted sugar baits (ATSBs) were proposed to control outdoor mosquitoes, including *Ae*. *albopictus* [14–18]. Sugar feeding is essential to the survival of both male and female mosquitoes. The ATSB strategy uses a bait with a sugar based lure to attract mosquitoes which are then killed when they ingest a toxicant in the bait. It has been proposed that attractive targeted sugar baits have the potential to be effective where vectors frequently feed on sugar or where natural sugar sources are limited [19]. Trials of ATSBs on *Ae*. *albopictus* in Israel and the United States demonstrated over 80% reductions in *Ae*. *albopictus* populations [15–18].

An initial study on Yorke Island found fructose prevalences of 36% and 28% in *Ae*. *albopictus* males and females, respectively [20]. The aim of this study was to quantify *Ae. albopictus* abundance and sugar feeding over time on different islands within the Torres Strait. This information is fundamental for understanding the potential of ATSBs to control *Ae. albopictus* across the islands beyond the cordon sanitaire.

## Materials and methods

### Study site

The study was conducted in 4^th^ – 26^th^ March 2021 and 23^rd^ March – 14^th^ April 2022 in Hammond (10.5586° S, 142.2072° E) and Yorke (9.7510° S, 143.4114° E) Islands in the Torres Strait, Australia [21]. Hammond Island (traditionally known as Kirriri) is a granite island formed from volcanic rocks located 6 km northwest of Thursday Island. The island is hilly and varies in altitude to 155 m above sea level (asl) [22] with a narrow coastal strip along the east coast where ~250 residents live [23]. The 1,607 ha area is dominated by bushland, including vine forests, coastal habitats, woodlands and paperbark open forests [22].

Yorke Island (traditionally known as Masig) is a coral cay located 160 kms northeast of Thursday Island. The topography of the 479 ha island is flat, between 0–3 m asl [24]. The 251 residents of Yorke Island mainly live on the northeastern coast [23]. The dominant habitats are beaches, sand flats and isolated fragmented bushland patches of scrub habitat [24]. The annual rainfall from 2019 to 2023 in Hammond and Yorke Islands averaged 1,749.4 mm and 1,036.1 mm, respectively [25].

### Field study

The study was conducted during the wet seasons, in March 2021 and April 2022. Mosquitoes were collected from both village and bushland habitats. Village habitats were defined as small residential settlements. The households usually had large yards or gardens near natural habitats or woodlands. The bushland habitats were defined as vast open areas dominated by trees and shrubs, used intermittently by humans. On each island, repeated sampling was conducted at eight fixed stations, with 4 stations in village and 4 stations in bushland habitats. The distance between stations was at least 100 meters. Additionally, stations in bushland habitats were at least 20 meters from roads or open spaces. A minimum 200 meters buffer zone separated village and bushland habitats to minimise mosquito populations mixing between the habitats (S1 Fig).

The vegetation at each sampling station was surveyed using a 2 m by 20 m belt transect divided into 10 quadrats (2 x 2 m). Surveys of each quadrat were divided into 4 squares (1 x 1 m). In each quadrat, the following parameters were estimated by direct observation: (1) the abundance and composition of plant specimens, (2) the prevalence of flowers, (3) the prevalence of fruit, and (4) canopy cover.

Parameter estimates were made in each square and then totalled for each quadrat. Plant abundance was estimated by direct counts. The prevalence of grasses, small herbs, seedlings, fruits, and flowers was categorised as absent, low, medium or high. Presence was assessed in each square and scored with a value of 0.25 if present or 0 if absent. The highest possible value in a single quadrat (of 4 squares) was 1, with a maximum potential value possible for a belt transect being 10. The values were summed within each transect and categorised as: absent (0), low (1–4), medium (5–7) and high (8–10). Plants were identified to family level and, if possible, to genus or species based on the distinctive morphology of leaves, flowers, and fruits. Photographs and some plant parts were collected to assist with later identifications in the field laboratory. Plants were identified using a range of methods including specimen collection, information from local communities and photographs before confirmation through dichotomous keys, spatial databases (i.e., Atlas of Living Australia) and later consultation with botanists [26,27]. Canopy cover was estimated by measuring the distance from each belt transect centre to where the canopy cover started and ended in centimeters. The calculation of canopy cover was defined as a percentage of the belt transect and assigned as one of four categories: absent (no canopy cover), low (≤ 50% canopy cover), medium (>50% – ≤ 85%) and high (>85%).

Mosquitoes were collected twice daily by sweep net between 07:00 and 10:00 and again between 15:00 and 18:00 for eight days in each wet season with the order of stations sampled rotated in a balanced design each day. Sweep net sampling was conducted for 10 minutes at each station using a 38-cm-diameter sweep net treated with a 1.0 g/kg esbiothrin, 0.3 g/kg permethrin, 0.2 g/kg imiprothrin (Mortein) [28]. Daily captured mosquitoes from each station were pooled in individual 1 L clear containers labelled with station identifier and collection time.

### Laboratory studies

#### Sample storage.

Twice daily, between 07:00 and 10:00 mosquitoes were collected in the morning and again between 15:00 and 18:00 mosquitoes were collected in the afternoon. Immediately following collection, captured mosquitoes were transported to a field laboratory and identified by morphology to species, sex and abdominal sugar fed status determined by examination using a stereomicroscope at 20x magnification [29]. *Aedes albopictus* were pooled by sex with a maximum of 10 specimens placed in 1.5 ml Eppendorf tubes labelled with collection date and station identifier. Samples were dried in a dry-heat bath at 100˚C for 60 minutes (with the lid open) before storing at room temperature with silica gel [20].

#### Cold anthrone test.

Fructose was quantified using a modified cold anthrone test in 96-well plates (BD Falcon, Germany) [20,30–32]. Each 96-well plate included field collected specimens, six duplicate standard fructose samples (ranging from 250 to 10 µg in 25% ethanol), a blank of 25% ethanol, two positive controls, and six negative controls. Control mosquitoes were adult *Ae. albopictus* reared from stage IV larvae and pupae collected in the study sites. Negative controls were adults within 24 hr of emergence that were deprived of sugar before heat fixed. Positive controls were emerged adults given access to a 10% fructose solution for 24 hr after emergence and then heat fixed (as above). Up to three *Ae. albopictus* were selected from each station and time point for analysis.

Each mosquito was mixed with 50 µL of 2% sodium sulphate and homogenised by a 3 mm glass bead (Sigma-Aldrich, USA) in Tissue-Lyser II (Qiagen, USA) at 30 Hz for 2 min. Then, 375 μL of chloroform:methanol solution (1:2 ratio) was added, and the mixture was centrifuged for 15 min at 1500 rpm. Next, 50 µL of supernatant from each sample was added to a microplate well. Under a fume hood, the samples evaporated overnight at room temperature. The next day, 200 µl of anthrone solution (containing 28.15% distilled water, 71.7% sulphuric acid and 0.15% anthrone) was added to each well and mixed. The plate was covered with aluminium foil and incubated for 75 min at room temperature. The optical densities (OD) of the samples were then measured using a microplate reader at 630 nm (POLARstar Omega; BMG Labtech, Australia).

#### Determining presence and amount of fructose in *Ae. albopictus.*

Fructose prevalence estimated the proportion of mosquitoes that had recently taken a sugar meal. The amount of fructose in the mosquito’s mid-gut indicates the size of sugar meals. As fructose digestion is rapid, the amount of fructose rapidly changes, and the amount of sugar provides an indication of when sugar feeding occurred. Thus, prevalence is important to facilitate comparisons of sugar feeding rates with other geographic areas. The OD value generated in the cold anthrone test was used to estimate the presence and amount of fructose in *Ae. albopictus* extracts. A mosquito was considered fructose-positive if its absorbance value exceeded the sum of the mean of the absorbance values from the six negative controls on each plate plus three standard deviations (cut-off = x ± 3 SD).

To calculate the amount of fructose (µg) in a positive mosquito, the OD value of the blank was subtracted from the sample absorbance and then divided by the slope of the standard curve [20]. As the extract assayed was 12% of the total extract volume the fructose content of the whole mosquito was calculated by multiplying the µg of fructose in the sample by 8.5.

### Statistical analysis

All fieldwork data, including vegetation surveys and mosquito collections were recorded using the Ona platform (http://ona.io). Cold anthrone test results were recorded using MS Excel (Microsoft Crop., USA). All statistical analyses were executed with the R statistical environment (v4.4.1), and all graphs were visualized using GraphPad (v10.2.2). Initial descriptive statistics of the vegetation differences across the two islands were compared with a Chi-squared test for flower and/or fruit presence and a t-test for canopy cover. The rare plant families which were encountered 5 or less times were removed from the dataset. The influence of plant families on the prevalence of flowers and/or fruits was compared with a GLM. To explore the relationship between the proportion of sugar-positive mosquitoes and the presence of flowers and/or fruit by plant family, we performed a correspondence analysis (CA), using the *ca* package in R. The CA method decomposes associations into principal dimensions, allowing visualization of the most influential components in the dataset and was most suitable for the multidimensional binomial dataset.

Principal Component Analysis (PCA) was conducted to explore the multivariate structure of plant composition data in the study. The dataset was centred and scaled to ensure uniformity among variables before PCA was applied to extract principal components explaining the dataset’s variance. Principal components were interpreted using loadings to understand relationships between plant families and components, using the *vegan* package in R. The significance of each PC was assessed using eigenvalues and scree plots. Scores of individual observations of significant PCs were used to visualize and interpret patterns of plant composition variation across sampling sites and habitats.

Generalised linear mixed models (GLMMs) were employed to assess the abundance of *Ae*. *albopictus* (negative binomial model), prevalence of fructose (binomial model) and fructose content (µg) per individual mosquito (gaussian model). These analyses were conducted with the *glmmTMB* package in R [33]. Random factors were collection date, sampling stations and study sites, with independent factors being sex, collection time, flower and fruit prevalence, canopy cover and vegetation PC values. A forward regression approach identified the most significant independent variables influencing the dynamics of the dependent variable. Each potential independent variable was evaluated individually to improve model fit, measured by Akaike’s Information Criterion (AIC). The comparative strength of evidence for each alternative model was compared using Akaike weights (*w*AIC), with *w*AIC values ranging from 0 (no support) to 1 (complete support), indicating the likelihood of each model being the best predictor of the data [34,35]. The forward regression process was continued until no additional independent variable significantly decreased the AIC. During the model development process, predictors were examined to minimize potential multicollinearity by ensuring predictors were conceptually distinct and not highly correlated based on exploratory assessments. The use of AIC for model selection provided an objective criterion to balance model fit and parsimony, reducing overfitting and aiding in selecting models that generalize well.

## Results

### Description of vegetation

Overall, 57 plant families were identified across Hammond and Yorke Islands with 43 plant families found on Hammond Island and 35 plant families found on Yorke Island (S1 Table). The average family richness per station was 12.5 (±1.13, SEM) and 8.5 (±1.18) on Hammond and Yorke Islands. In terms of habitat types, the average family richness was 11.3 (±0.96) in villages and 9.75 (±1.66) in bushland habitats. Fabaceae (or Leguminosae) and Combretaceae were the most abundant plant families on both islands with *Terminalia catappa* the most frequently recorded plant species.

On Hammond Island, the predominant plant life forms were seedlings (28%), trees (26%) and herbs (24%). Conversely, on Yorke Island, the most common plant life forms were shrubs (26%), followed by seedlings (19%), herbs (16%) and trees (15%). While village habitats were dominated by herbs (38%) and seedlings (12%), bushland habitats exhibited more balance in plant life forms with trees and seedlings commonly found in both habitat types (S2 Fig).

Overall, 40% of plant occurrences had some form of flowers (even at a low flowering density) and 19% had fruits, and the presence of any potential sugar source (flowers and/or fruit) was analysed as a single category. During the vegetation survey, the prevalence of either flowers and/or fruits (χ^2^ = 46.11, df = 1, p < 0.001) was associated with island, with both being more prevalent on Yorke Island (S3 Fig). The average canopy coverage was 90% and there was no difference between islands (t = −0.889, df = 14, p-value = 0.3885). Village habitats had more flowers and/or fruit than bushland habitats (χ^2^ = 53.99, df = 1, p < 0.001).

The presence of flowers and/or fruit varied significantly across plant families (χ^2^ = 95.97, df = 13, p < 0.001). Certain families had notably higher flower and/or fruit prevalence compared with the reference, being: Fabaceae, Asteraceae, Euphorbiaceae, Phyllanthaceae and Poaceae (S4 Fig). This was mostly driven by flower presence.

### *Aedes albopictus* abundance

**Fig 1:**
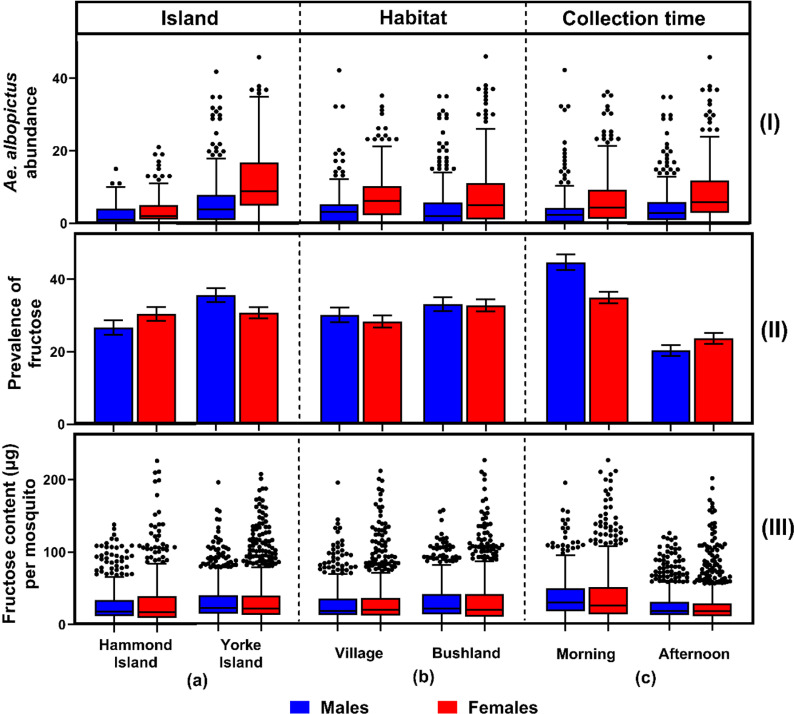
(I) *Aedes albopictus* abundance, (II) prevalence of fructose, and (III) fructose content (µg) per *Ae. albopictus* by (a) island, (b) habitat, and (c) collection time.

**Fig 2:**
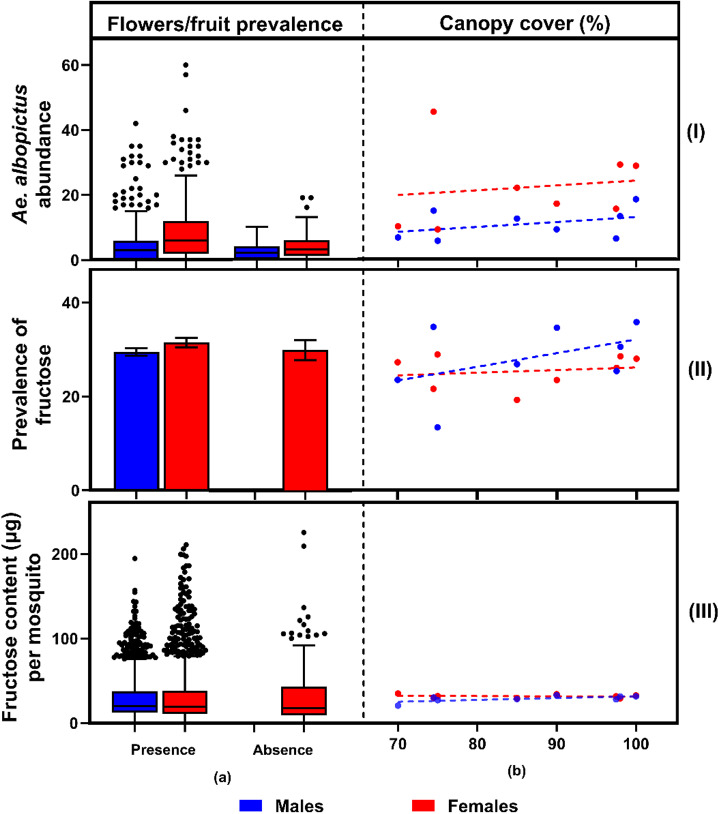
(I) *Aedes albopictus* abundance, (II) prevalence of fructose, and (III) fructose content (µg) per *Ae. albopictus* by (a) flowers/fruit prevalence and (b) canopy cover (%).

A total of 6,186 *Ae. albopictus* were captured by sweep net on Hammond and Yorke Islands, with 4,007 (64.8%) being females (Fig 1(I)). *Aedes scutellaris* was also collected on both islands (n = 37), but *Ae. aegypti* was not captured. The results from the GLMM showed that *Ae. albopictus* abundance was significantly associated by island, sex, collection time, canopy, PC2 (variation in vegetation gradients), habitat and the presence of flowers and/or fruits (GLMM Table 1 and Figs 1(I) and 2(I)). The abundance of male *Ae. albopictus* was consistently less than female abundance across the different islands, habitats, collection times, flower and/or fruit presence, and canopy cover (Figs 1(I) and 2(I)).

**Table 1:** GLMM with forward regression nested model evaluation of best predictors for *Ae. albopictus* abundance, the prevalence of fructose-positive *Ae. albopictus* and fructose quantity (µg). The model comparison was made on the basis of ΔAIC, wAIC, chi-square and *P*-value. The explanatory variables were sex, collection time, island, habitat, flowers, and fruit prevalence.

Model	Variable entered	*df*	AIC	ΔAIC	*w*AIC	*X2*	*P* value
*Aedes albopictus* abundance							
1	Island	5	5597.3	182.40	0.00	210.22	<0.001
2	Island + Sex	6	5521.1	106.21	0.00	78.21	<0.001
3	Island + Sex + Time	7	5472.9	58.01	0.00	50.22	<0.001
4	Island + Sex + Time + Canopy	8	5447.6	32.77	0.00	27.26	<0.001
5	Island + Sex + Time + Canopy + PC2	9	5422.3	7.45	0.02	27.35	<0.001
6	Island + Sex + Time + Canopy + PC2 + Habitat	10	5417.6	2.80	0.19	6.69	0.009
7	Island + Sex + Time + Canopy + PC2 + Habitat + FlowersFruits	11	5414.7	0.00	0.79	4.83	0.027
Prevalence of fructose							
1	Time	5	1714.0	9.56	0.01	98.31	<0.001
2	Time + Sex	6	1706.7	2.19	0.25	9.39	0.002
3	Time + Sex + PC2	7	1704.4	0.00	0.74	4.22	0.040
Fructose (µg) per *Aedes albopictus*							
1	Time	5	7690.8	32.45	0.00	119.73	<0.001
2	Time + Sex	6	7674.6	16.25	0.00	18.20	<0.001
3	Time + Sex + Island	7	7659.5	1.20	0.35	17.06	<0.001
4	Time + Sex + Island + FlowersFruits	8	7658.3	0.00	0.65	3.21	0.073

### Prevalence of fructose

**Fig 3:**
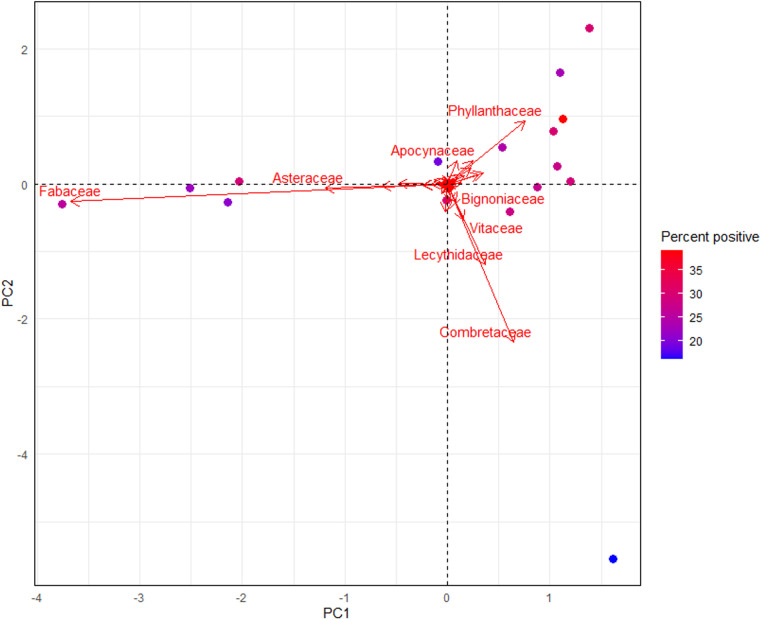
Principal component analysis (PCA) of plant family composition variation across sampling sites (islands) and habitats.

Overall, 2,641 *Ae. albopictus* were tested for fructose. The prevalence of fructose was significantly associated by collection time, sex and PC2 (Table 1 and Fig 1(II)). Fructose positive male *Ae. albopictus* were more prevalent overall than female fructose positives (31.6% ± 1.4 in males and 30.5% ± 1.2 in females; GLMM, *P* = 0.002, Figs 1(II) and 2(II)). Fructose positive *Ae. albopictus* were almost two times more prevalent in the morning than in the afternoon (42.0% ± 1.9 in morning and 22.0% ± 1.9 in afternoon; GLMM, *P* < 0.001). Principal Component 2, derived from the PCA of plant composition data, exerted a notable influence on fructose prevalence, suggesting that specific combinations of plant species were correlated with variations in fructose positivity. The plant family Phyllanthaceae was strongly associated with PC2, suggesting plant communities dominated by this family were correlated with an increased prevalence of fructose positive *Ae. albopictus* (Fig 3, loading PC1: 0.7569, PC2: 0.9312). As noted above, Phyllanthaceae was one of the families that had significantly higher flower and/or fruit presence.

The correspondence analysis identified distinct relationships between the proportion of sugar-positive mosquitoes and the presence of flowers by plant family across the study sites. The first two principal dimensions accounted for a cumulative inertia of 52.5% (28.2% for Dimension 1 and 24.3% for Dimension 2). Dimension 1 likely represents broad patterns of differentiation among plant families, while Dimension 2 captures more specific associations within these broad patterns. Among plant families, significant associations with the principal dimensions were observed. For example, the Phyllanthaceae family exhibited the highest contribution to Dimension 1 (“ctr”: 768), driven by its widespread presence and association with sugar-positive mosquitoes (S5 Fig).

### Fructose (µg) per *Aedes albopictus*

The average fructose-positive *Ae. albopictus* had 62.9 µg (± 1.4) fructose, with an average cut-off value of 15.9 µg (± 0.3). The amount of fructose per mosquito was significantly associated by sex, collection time, and island; with the addition of flower and/or fruit presence almost significant (Table 1 and Fig 1(II)). Overall, females took larger fructose meals than males (31.2 µg ± 0.9 in females; 29.4 µg ± 0.8 in males; GLMM, *P* < 0.001). The average fructose amount in *Ae. albopictus* collected in the morning was almost 60% more than that found in the afternoon (38.48 µg ± 1.1 in the morning and 24 µg ± 0.7 in the afternoon; GLMM, *P* < 0.001). The amount of fructose found in *Ae albopictus* on Yorke Island was higher than on Hammond Island (31.5 µg ± 0.8 in Yorke Island; 29.1 µg ± 1.0 in Hammond Island, GLMM, *P* = 0.021).

## Discussion

The distribution of *Aedes* in the Torres Strait Islands changed over the past three decades. Following dengue control efforts, including residual spraying in domestic surface containers and the removal of rainwater tanks and wells in 1997, *Ae. aegypti* was displaced by *Ae. scutellaris* [1,36]. When *Ae. albopictus* was introduced from Indonesia in 2005, the distribution and abundance of both *Ae. scutellaris* and *Ae. aegypti* diminished while *Ae. albopictus* became the predominant *Aedes* species across the Torres Strait Islands [11,37].

In this study, male *Ae. albopictus* were less frequently collected than females which can be attributed to the collection method as well as behavioural and lifespan differences. The sweep net technique indirectly uses the collector body as bait to attract blood meal seeking female mosquitoes and collaterally captures males seeking females for mating [38]. In general, the survivorship of adult males is shorter than females, which reduces male abundance and therefore lowers the ratio of male to female mosquitoes [39].

Sex and collection time were the strongest factors influencing both the amount and prevalence of fructose in *Ae. albopictus* in the Torres Strait Islands. The prevalence of fructose in both sexes was highest in the morning, indicating that morning may be the preferred sugar-feeding time for *Ae. albopictus*. This aligns with known mosquito behaviour, as sugar feeding often occurs during periods of reduced activity when energy demands are lower. Female *Ae. albopictus* exhibited a lower prevalence of fructose compared to males; a pattern consistent with other mosquito species [19,30,40,41]. While both sexes sugar feed for energy, females also obtain energy from blood meals, reducing their dependence of sugar feeding [30,42,43]. Moreover, a fructose meal is rapidly digested in a mosquito’s abdomen, being only detectable for 48 hours after feeding for *Ae. albopictus* and *Ae. aegypti*, depending on factors such as environmental conditions, sugar concentration, and mosquito metabolic rates. As such, the cold anthrone test detects only recent sugar meals. Females, due to their larger body size and the energy requirements for reproduction, consumed larger sugar meals when they sugar fed. Larger sugar meals could result in fructose being detectable for a longer duration in females than in males.

Plant communities that were dominated by species from the Phyllanthaceae family were positively correlated with higher abundance and fructose prevalence of *Aedes albopictus*. During the surveys, the Phyllanthaceae family was one of the families with a higher occurrence of flowers and/or fruit. This plant family was only present on Hammond Island, mostly in bushland areas and comprised three species: *Cleistanthus* sp. (most common), *Glochidion* sp., and *Bridelia* sp. The *Cleistanthus* sp. produces small and often inconspicuous flowers and fruits. While the flowers aren’t conspicuous and may not produce abundant nectar, there are some limited reports that the flowers are still attractive to small insects. The positive correlation between fructose prevalence and PC2, as opposed to the overall presence of flowers and/or fruit in the environment indicates that the composition of the vegetation structure does have importance. During the vegetation surveys, fruits were infrequently observed while flowers were present at all but one station, albeit often in low densities. This suggests that the overall measure of flower and/or fruit presence is not nuanced enough to represent the cues that to which the insect population likely responds. The correspondence analysis (ca) highlights meaningful ecological relationships between mosquito sugar-feeding behaviors and local floral resources. These results underscore the need to investigate the role of specific plant families in shaping vector ecology and disease transmission risk.

Experimental trials suggest that ATSBs are a promising approach for controlling outdoor mosquitoes. In a trial conducted in Haifa, Israel, where fructose prevalence prior to ATSB deployment was 68% and 63% in male and female *Ae. albopictus*, *Aedes albopictus* abundance was reduced by 84% in mosquitoes sampled by human landing catches following implementation of ATSBs [16]. Similarly, following deployment of ATSBs in a desert oasis where 72% of *Anopheles sergentii* were positive for sugar, *An. sergentii* populations were reduced by 97.5% [44].

Defining the level of sugar in the environment is complex and authors have used different methods, including estimating the percentage of flowering vegetation or a measure of landscape greenness. Here the percentage of flowering vegetation ranged widely across the sample stations and an overall average of 40% of vegetation occurrences having flowers. An ATSB treatment trial in Bamako, Mali effectively reduced *Ae. aegypti* abundance by approximately 95% in both sugar-rich (50% flowering vegetation) and sugar-poor (<5% flowering vegetation) environments [19], showing that ATSBs were effective in environments within a wide range of natural sugar sources. In Mali, this fructose prevalence was similar to what was found in *Ae albopictus* in this study (24–46% in males and 24–32% in females) and a previous study on Yorke Island (35% in males and 28% in females) [20]. This comparable fructose prevalence seen in Mali and in the Torres Strait Islands suggests that ATSB trials would be warranted to determine their potential to reduce the risk of *Ae. albopictus* mosquitoes being introduced into the cordon sanitaire. This, in turn, would lower the chances of *Ae. albopictus* spreading to mainland Australia via transport hubs on Horn and Thursday Islands.

## Conclusion

Understanding the sugar feeding behaviour of *Aedes albopictus* provides fundamental information for assessing the potential of ATSB applications to control this outdoor mosquito. Mosquito sex and collection time were parameters associated with the abundance of *Ae. albopictus*, the fructose prevalence and the amount of fructose in *Ae. albopictus*. Male and female *Ae. albopictus* harboured different sugar amounts and the prevalences of sugar positives by sex varied by time of day. The comparable fructose prevalence seen in Mali and in the Torres Strait Islands suggests that ATSBs trials would be warranted to determine their potential to both control *Ae. albopictus* and in so doing reduce the risk of *Ae. albopictus* mosquitoes escaping from the cordon sanitaire to the mainland of Australia.

## Supporting information

S1 Table List of plant families at each station on Hammond and Yorke Islands. “1” denotes present and “0” denotes absent during the survey.
(DOCX)

S1 Fig Distribution of stations on Torres Strait Island. (a) Hammond and (b) Yorke Islands. The basemap was created with Esri World Imagery (WGS84) https://www.arcgis.com/home/item.html?id=52bdc7ab7fb044d98add148764eaa30a
(TIFF)

S2 Fig Vegetation life-forms. (a) Hammond Island; (b) Yorke Island; (c) Village habitat; (d) Bushland habitat.
(TIF)

S3 Fig Prevalence of flowers and/or fruit, and percentage of canopy cover by islands and habitat types. (TIF)

S4 Fig Prevalence of flowers and/or fruit by common plant families. (TIF)

S5 Fig Correspondence Analysis (CA) plot illustrating the relationship between plant families and their presence in flower and fruit occurrence. The analysis identifies patterns of association between locations and plant families based on flower and fruit presence, providing insights into the floristic composition and reproductive traits across the surveyed areas.
(TIF)
