# Supplementary material for: Sugar feeding by Aedes albopictus in the Torres Strait, Australia
Source: PLoS Negl Trop Dis. 2025 Feb 7;19(2):e0012856. doi: 10.1371/journal.pntd.0012856 (PMC11819548; doi:10.1371/journal.pntd.0012856)
Supplement: S1 Table — “1” denotes present and “0” denotes absent during the survey. (DOCX) [file pntd.0012856.s001.docx]

# Supporting Information

**Sugar feeding by *Aedes albopictus* in the Torres Strait, Australia**

Boni F. Sebayang, Tanya L. Russell, Susannah Mosby, Richard Gela, Darcy L. Roeger, Bram van de Straat, Kyran M. Staunton, Thomas R. Burkot

**S1 Table. List of plant families at each station on Hammond and Yorke Islands.** “1” denotes present and “0” denotes absent during the survey.

| **No.** | **Plant family** | **Hammond** | **Yorke** |  | **No.** | **Plant family** | **Hammond** | **Yorke** |
| --- | --- | --- | --- | --- | --- | --- | --- | --- |
| 1 | Amaryllidaceae | 1 | 0 |  | 31 | Menispermaceae | 1 | 0 |
| 2 | Anacardiaceae | 1 | 1 |  | 32 | Mimosaceae | 0 | 1 |
| 3 | Apocynaceae | 1 | 1 |  | 33 | Moraceae | 1 | 0 |
| 4 | Araceae | 1 | 1 |  | 34 | Musaceae | 1 | 0 |
| 5 | Arecaceae | 1 | 1 |  | 35 | Myristicaceae | 1 | 0 |
| 6 | Aristolochiaceae | 0 | 1 |  | 36 | Myrtaceae | 1 | 1 |
| 7 | Asparagaceae | 0 | 1 |  | 37 | Oleaceae | 1 | 0 |
| 8 | Asteraceae | 1 | 1 |  | 38 | Pandanaceae | 1 | 1 |
| 9 | Bignoniaceae | 1 | 1 |  | 39 | Passifloraceae | 0 | 1 |
| 10 | Burseraceae | 1 | 0 |  | 40 | Phyllanthaceae | 1 | 0 |
| 11 | Caesalpiniaceae | 1 | 0 |  | 41 | Pittosporaceae | 0 | 1 |
| 12 | Capparaceae | 1 | 0 |  | 42 | Poaceae | 1 | 1 |
| 13 | Casuarinaceae | 0 | 1 |  | 43 | Polygalaceae | 1 | 0 |
| 14 | Chrysobalanaceae | 1 | 0 |  | 44 | Polypodiaceae | 0 | 1 |
| 15 | Colchicaceae | 0 | 1 |  | 45 | Portulaceae | 1 | 0 |
| 16 | Combretaceae | 1 | 1 |  | 46 | Putranjivaceae | 0 | 1 |
| 17 | Commelinaceae | 1 | 1 |  | 47 | Rhamnaceae | 0 | 1 |
| 18 | Convolvulaceae | 0 | 1 |  | 48 | Rhizophoraceae | 1 | 0 |
| 19 | Crassulaceae | 0 | 1 |  | 49 | Rubiaceae | 1 | 1 |
| 20 | Dilleniaceae | 0 | 1 |  | 50 | Rutaceae | 1 | 1 |
| 21 | Dioscoreaceae | 1 | 0 |  | 51 | Sapindaceae | 1 | 0 |
| 22 | Elaeocarpaceae | 1 | 0 |  | 52 | Sapotaceae | 1 | 1 |
| 23 | Euphorbiaceae | 1 | 1 |  | 53 | Smilacaceae | 1 | 1 |
| 24 | Fabaceae | 1 | 1 |  | 54 | Taccaceae | 0 | 1 |
| 25 | Flagellariaceae | 1 | 1 |  | 55 | Turneraceae | 1 | 0 |
| 26 | Lamiaceae | 1 | 1 |  | 56 | Verbenaceae | 1 | 1 |
| 27 | Lauraceae | 1 | 0 |  | 57 | Vitaceae | 1 | 0 |
| 28 | Lecythidaceae | 1 | 0 |  | **Grand Total (df = 1)** | | **43** | **35** |
| 29 | Loranthaceae | 1 | 0 |  |  |  |  |  |
| 30 | Malvaceae | 1 | 1 |  |  |  |  |  |
