# Supplementary figures and images for: Sugar feeding by Aedes albopictus in the Torres Strait, Australia
Source: PLoS Negl Trop Dis. 2025 Feb 7;19(2):e0012856. doi: 10.1371/journal.pntd.0012856 (PMC11819548; doi:10.1371/journal.pntd.0012856)

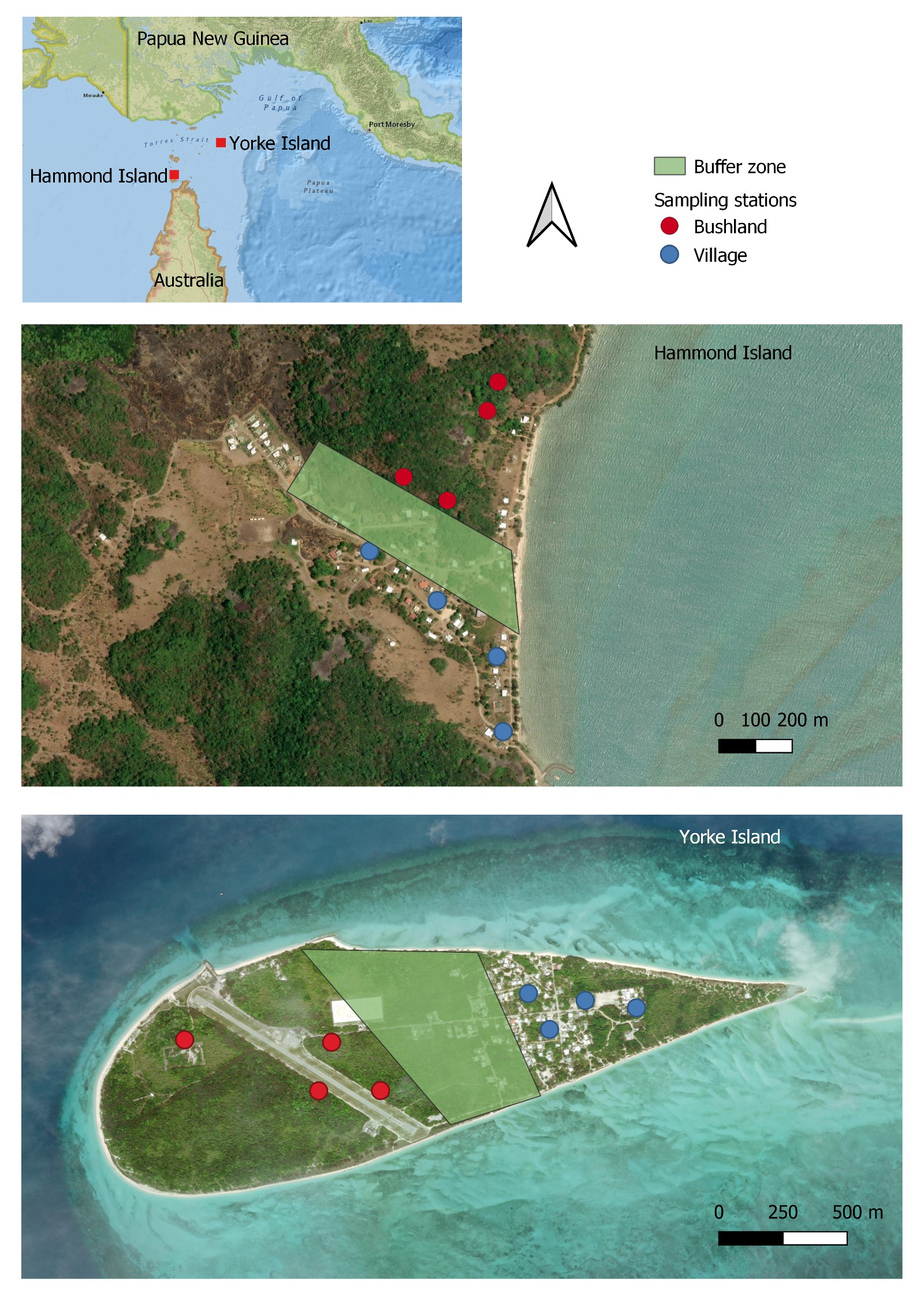

Supplement: S1 Fig — (a) Hammond and (b) Yorke Islands. The basemap was created with Esri World Imagery (WGS84) https://www.arcgis.com/home/item.html?id=52bdc7ab7fb044d98add148764eaa30a (TIFF) [file pntd.0012856.s002.tiff]

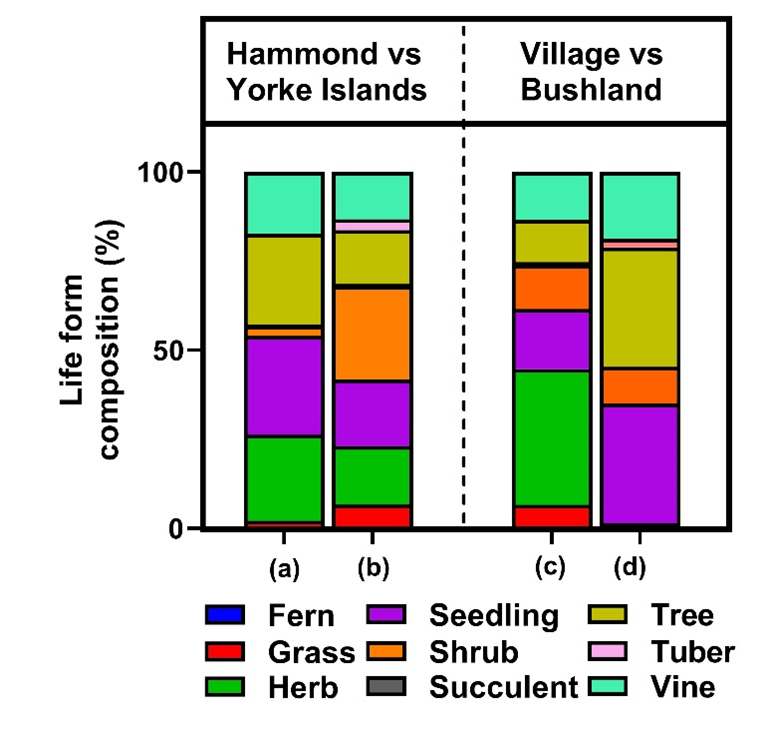

Supplement: S2 Fig — (a) Hammond Island; (b) Yorke Island; (c) Village habitat; (d) Bushland habitat. (TIF) [file pntd.0012856.s003.tif]

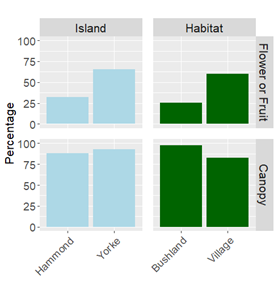

Supplement: S3 Fig — (TIF) [file pntd.0012856.s004.tif]

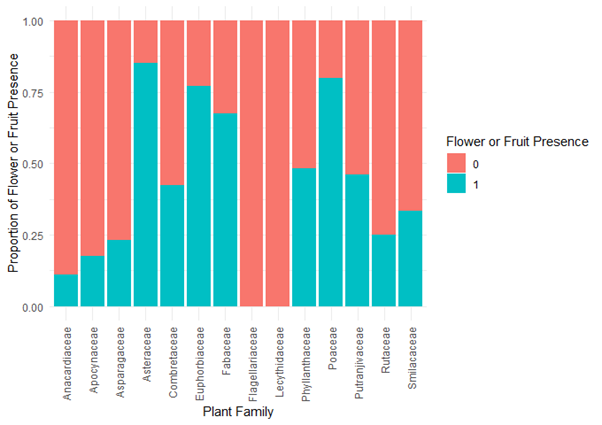

Supplement: S4 Fig — (TIF) [file pntd.0012856.s005.tif]

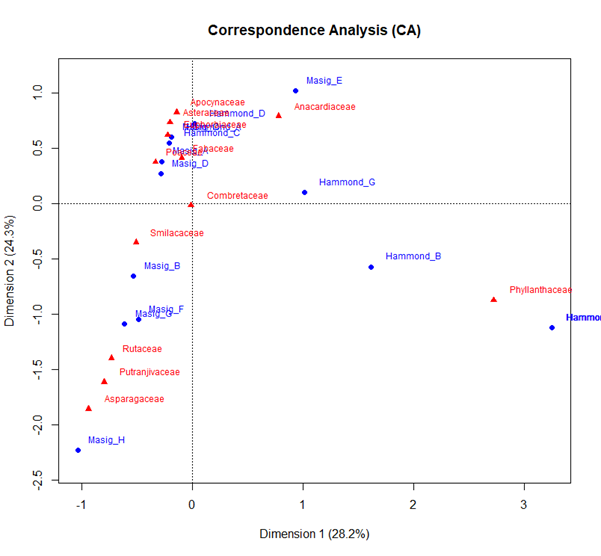

Supplement: S5 Fig — The analysis identifies patterns of association between locations and plant families based on flower and fruit presence, providing insights into the floristic composition and reproductive traits across the surveyed areas. (TIF) [file pntd.0012856.s006.tif]
